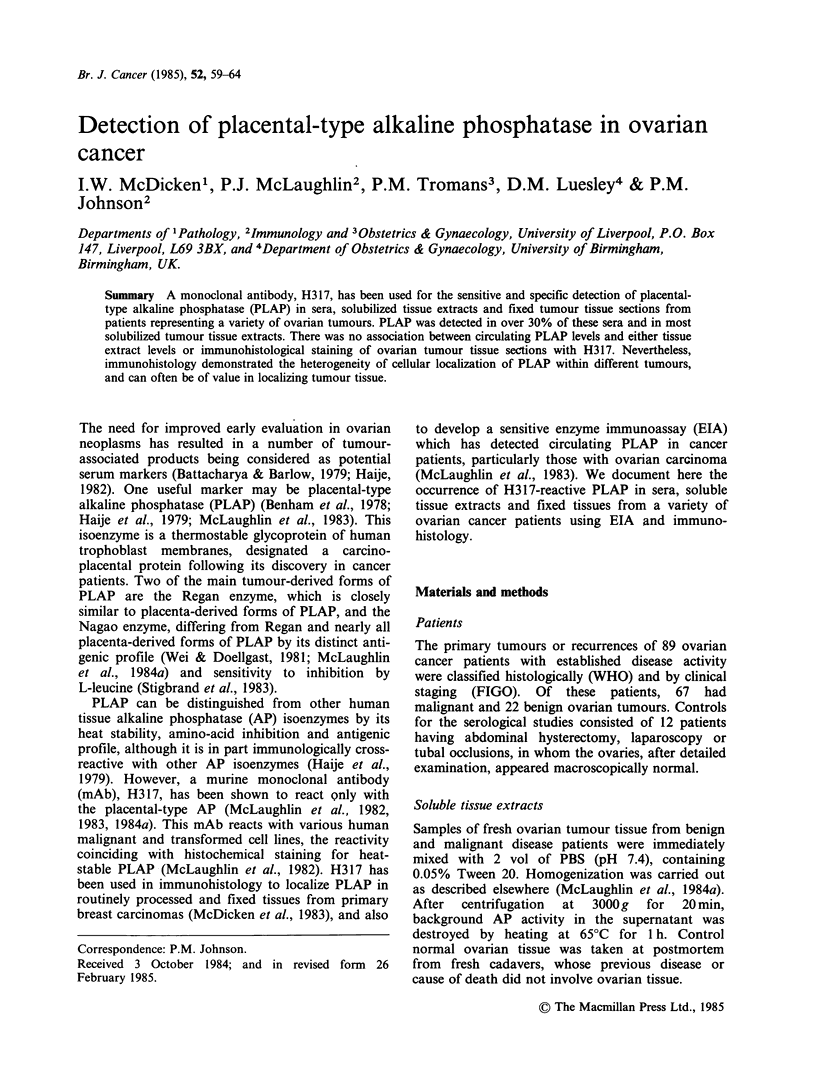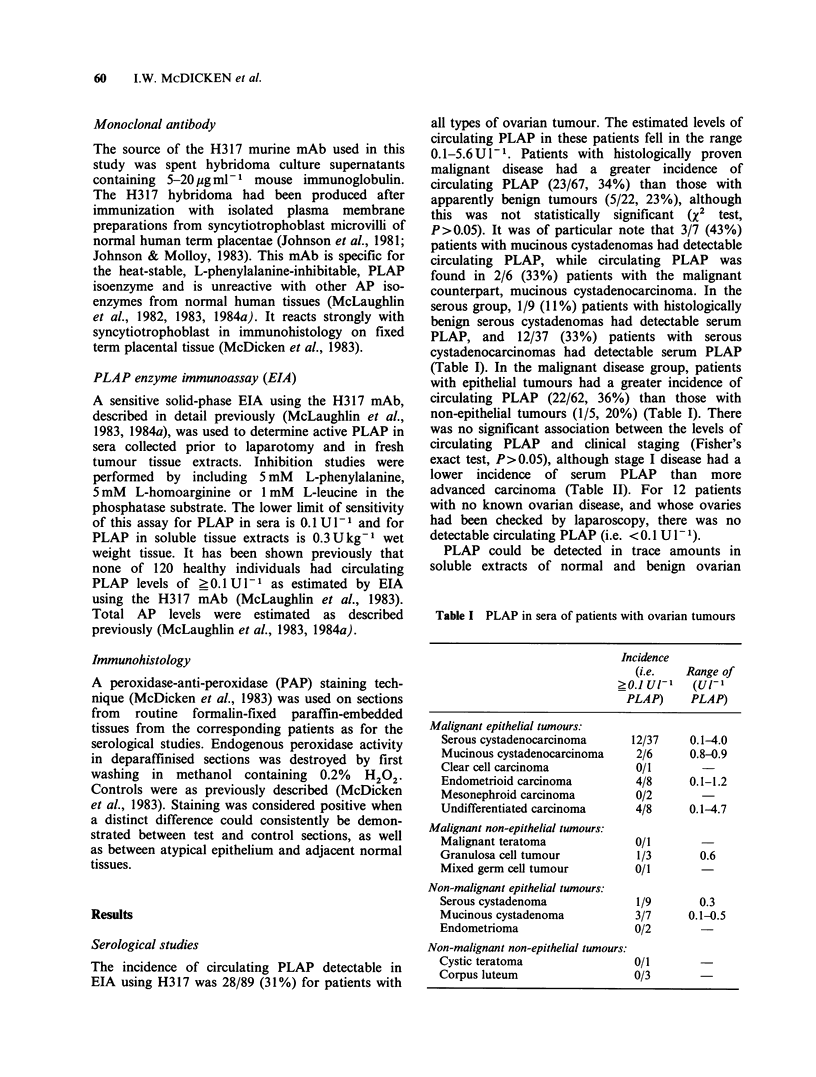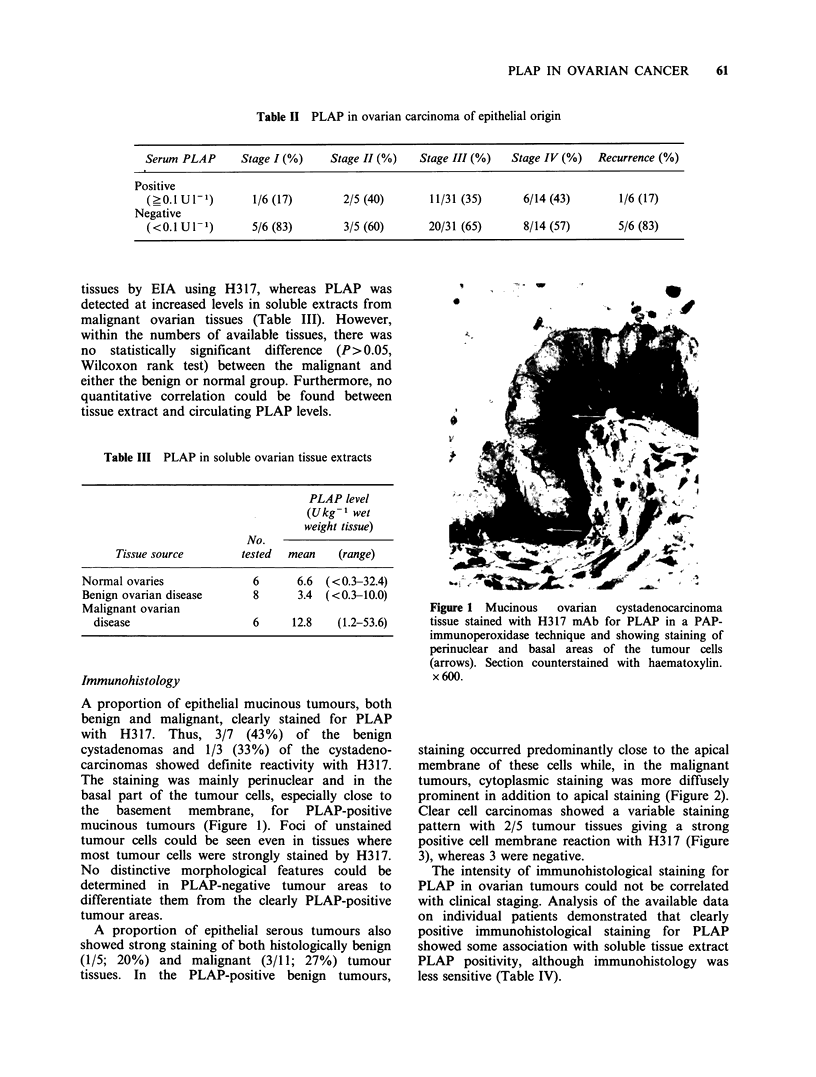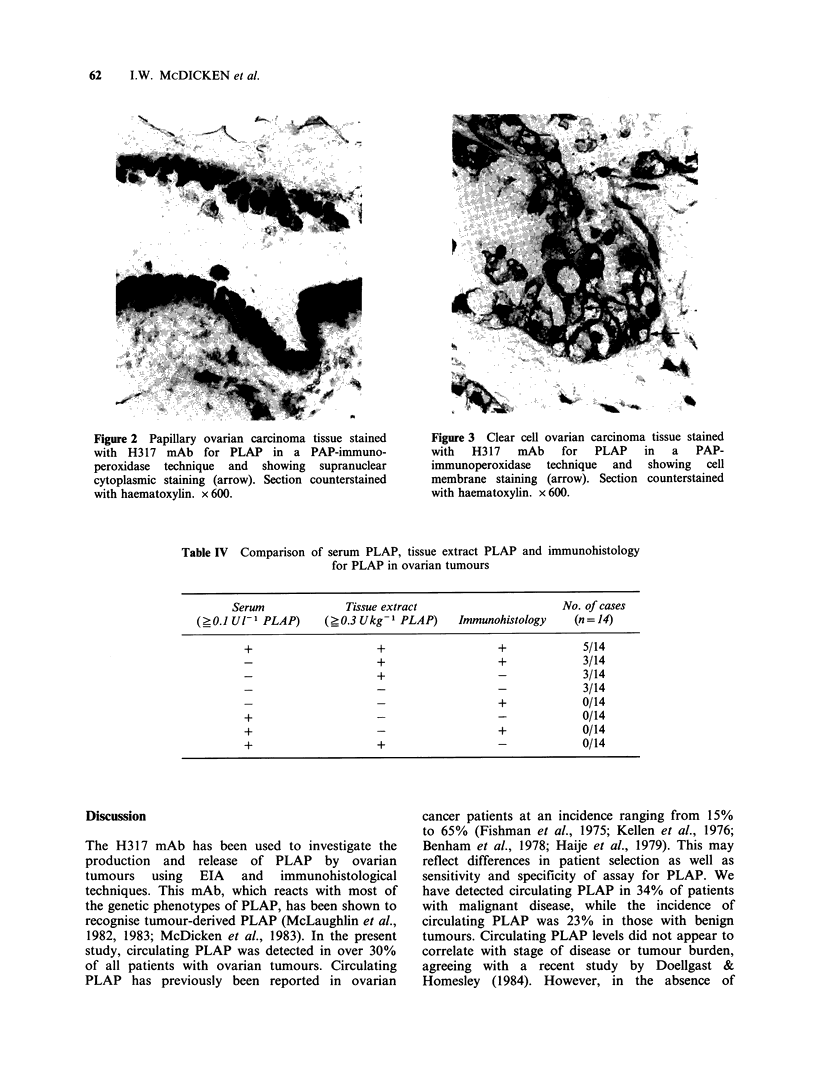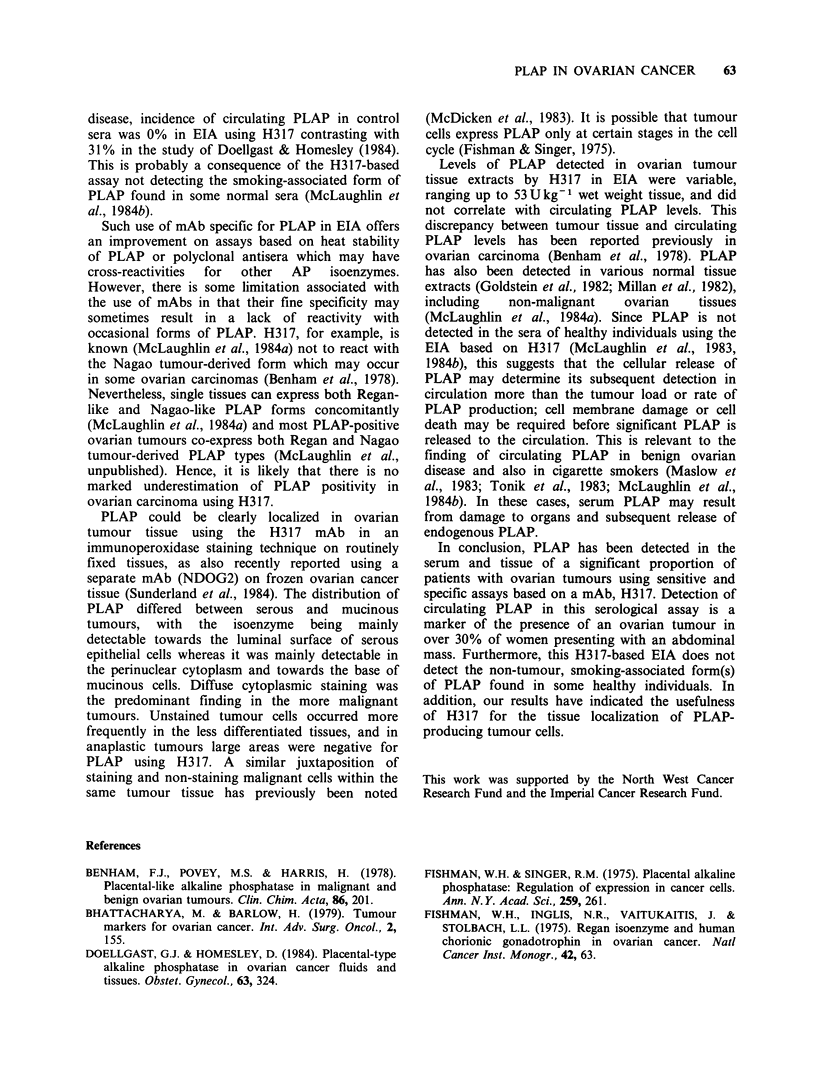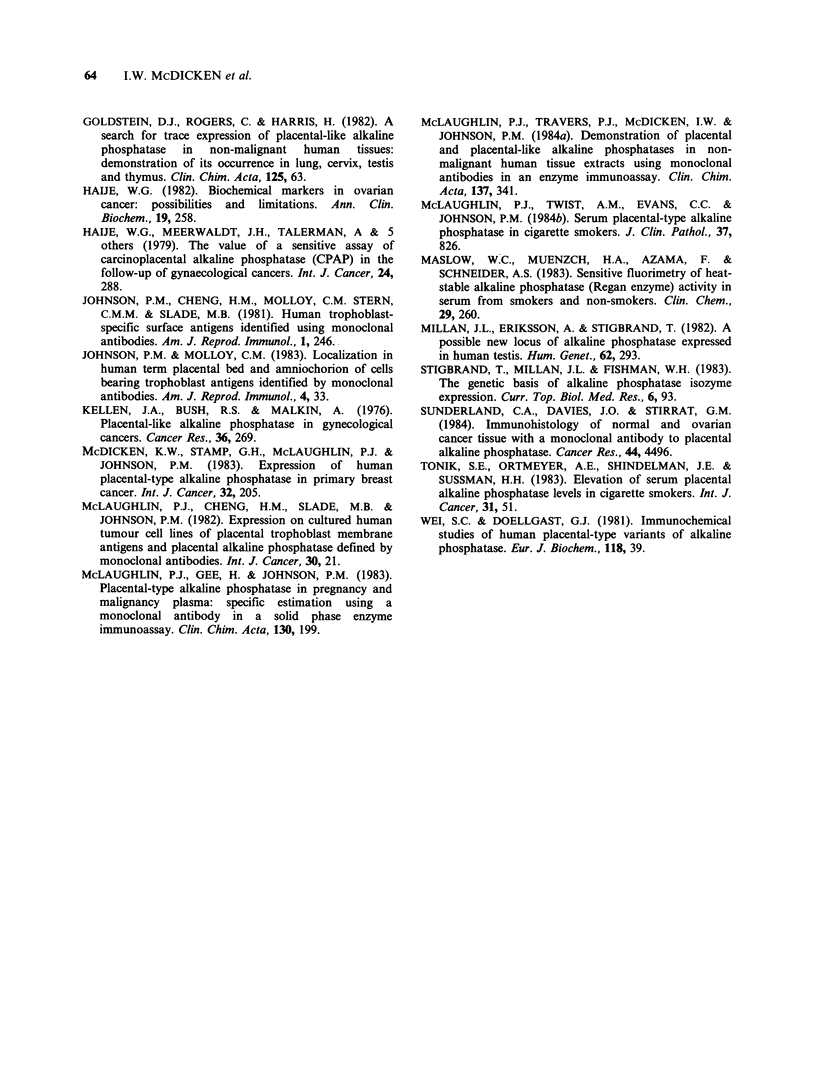# Detection of placental-type alkaline phosphatase in ovarian cancer.

**DOI:** 10.1038/bjc.1985.149

**Published:** 1985-07

**Authors:** I. W. McDicken, P. J. McLaughlin, P. M. Tromans, D. M. Luesley, P. M. Johnson

## Abstract

**Images:**


Br. J. Cancer (1985), **52**, 59–64

### Detection of placental-type alkaline phosphatase in ovarian cancer

I.W. McDicken[1], P.J. McLaughlin[2], P.M. Tromans[3], D.M. Luesley[4] & P.M. Johnson[2]

*Departments of [1]Pathology, [2]Immunology and [3]Obstetrics & Gynaecology, University of Liverpool, P.O. Box 147, Liverpool, L69 3BX, and [4]Department of Obstetrics & Gynaecology, University of Birmingham, Birmingham, UK.*

**Summary** A monoclonal antibody, H317, has been used for the sensitive and specific detection of placental-type alkaline phosphatase (PLAP) in sera, solubilized tissue extracts and fixed tumour tissue sections from patients representing a variety of ovarian tumours. PLAP was detected in over 30% of these sera and in most solubilized tumour tissue extracts. There was no association between circulating PLAP levels and either tissue extract levels or immunohistological staining of ovarian tumour tissue sections with H317. Nevertheless, immunohistology demonstrated the heterogeneity of cellular localization of PLAP within different tumours, and can often be of value in localizing tumour tissue.

The need for improved early evaluation in ovarian neoplasms has resulted in a number of tumour-associated products being considered as potential serum markers (Battacharya & Barlow, 1979; Haije, 1982). One useful marker may be placental-type alkaline phosphatase (PLAP) (Benham *et al.*, 1978; Haije *et al.*, 1979; McLaughlin *et al.*, 1983). This isoenzyme is a thermostable glycoprotein of human trophoblast membranes, designated a carcino-placental protein following its discovery in cancer patients. Two of the main tumour-derived forms of PLAP are the Regan enzyme, which is closely similar to placenta-derived forms of PLAP, and the Nagao enzyme, differing from Regan and nearly all placenta-derived forms of PLAP by its distinct antigenic profile (Wei & Doellgast, 1981; McLaughlin *et al.*, 1984*a*) and sensitivity to inhibition by L-leucine (Stigbrand *et al.*, 1983).

PLAP can be distinguished from other human tissue alkaline phosphatase (AP) isoenzymes by its heat stability, amino-acid inhibition and antigenic profile, although it is in part immunologically cross-reactive with other AP isoenzymes (Haije *et al.*, 1979). However, a murine monoclonal antibody (mAb), H317, has been shown to react only with the placental-type AP (McLaughlin *et al.*, 1982, 1983, 1984*a*). This mAb reacts with various human malignant and transformed cell lines, the reactivity coinciding with histochemical staining for heat-stable PLAP (McLaughlin *et al.*, 1982). H317 has been used in immunohistology to localize PLAP in routinely processed and fixed tissues from primary breast carcinomas (McDicken *et al.*, 1983), and also to develop a sensitive enzyme immunoassay (EIA) which has detected circulating PLAP in cancer patients, particularly those with ovarian carcinoma (McLaughlin *et al.*, 1983). We document here the occurrence of H317-reactive PLAP in sera, soluble tissue extracts and fixed tissues from a variety of ovarian cancer patients using EIA and immunohistology.

Correspondence: P.M. Johnson.

Received 3 October 1984; and in revised form 26 February 1985.

### Materials and methods

### *Patients*

The primary tumours or recurrences of 89 ovarian cancer patients with established disease activity were classified histologically (WHO) and by clinical staging (FIGO). Of these patients, 67 had malignant and 22 benign ovarian tumours. Controls for the serological studies consisted of 12 patients having abdominal hysterectomy, laparoscopy or tubal occlusions, in whom the ovaries, after detailed examination, appeared macroscopically normal.

### *Soluble tissue extracts*

Samples of fresh ovarian tumour tissue from benign and malignant disease patients were immediately mixed with 2 vol of PBS (pH 7.4), containing 0.05% Tween 20. Homogenization was carried out as described elsewhere (McLaughlin *et al.*, 1984*a*). After centrifugation at 3000 *g* for 20 min, background AP activity in the supernatant was destroyed by heating at 65°C for 1 h. Control normal ovarian tissue was taken at postmortem from fresh cadavers, whose previous disease or cause of death did not involve ovarian tissue.

© The Macmillan Press Ltd., 1985

*Monoclonal antibody*

The source of the H317 murine mAb used in this study was spent hybridoma culture supernatants containing 5–20 $\mu g\, ml^{-1}$ mouse immunoglobulin. The H317 hybridoma had been produced after immunization with isolated plasma membrane preparations from syncytiotrophoblast microvilli of normal human term placentae (Johnson *et al.*, 1981; Johnson & Molloy, 1983). This mAb is specific for the heat-stable, L-phenylalanine-inhibitable, PLAP isoenzyme and is unreactive with other AP isoenzymes from normal human tissues (McLaughlin *et al.*, 1982, 1983, 1984*a*). It reacts strongly with syncytiotrophoblast in immunohistology on fixed term placental tissue (McDicken *et al.*, 1983).

*PLAP enzyme immunoassay* (*EIA*)

A sensitive solid-phase EIA using the H317 mAb, described in detail previously (McLaughlin *et al.*, 1983, 1984*a*), was used to determine active PLAP in sera collected prior to laparotomy and in fresh tumour tissue extracts. Inhibition studies were performed by including 5 mM L-phenylalanine, 5 mM L-homoarginine or 1 mM L-leucine in the phosphatase substrate. The lower limit of sensitivity of this assay for PLAP in sera is 0.1 $U\, l^{-1}$ and for PLAP in soluble tissue extracts is 0.3 $U\, kg^{-1}$ wet weight tissue. It has been shown previously that none of 120 healthy individuals had circulating PLAP levels of $\geq 0.1\, U\, l^{-1}$ as estimated by EIA using the H317 mAb (McLaughlin *et al.*, 1983). Total AP levels were estimated as described previously (McLaughlin *et al.*, 1983, 1984*a*).

*Immunohistology*

A peroxidase-anti-peroxidase (PAP) staining technique (McDicken *et al.*, 1983) was used on sections from routine formalin-fixed paraffin-embedded tissues from the corresponding patients as for the serological studies. Endogenous peroxidase activity in deparaffinised sections was destroyed by first washing in methanol containing 0.2% $H_2O_2$. Controls were as previously described (McDicken *et al.*, 1983). Staining was considered positive when a distinct difference could consistently be demonstrated between test and control sections, as well as between atypical epithelium and adjacent normal tissues.

### Results

*Serological studies*

The incidence of circulating PLAP detectable in EIA using H317 was 28/89 (31%) for patients with all types of ovarian tumour. The estimated levels of circulating PLAP in these patients fell in the range 0.1–5.6 $U\, l^{-1}$. Patients with histologically proven malignant disease had a greater incidence of circulating PLAP (23/67, 34%) than those with apparently benign tumours (5/22, 23%), although this was not statistically significant ($\chi^2$ test, $P > 0.05$). It was of particular note that 3/7 (43%) patients with mucinous cystadenomas had detectable circulating PLAP, while circulating PLAP was found in 2/6 (33%) patients with the malignant counterpart, mucinous cystadenocarcinoma. In the serous group, 1/9 (11%) patients with histologically benign serous cystadenomas had detectable serum PLAP, and 12/37 (33%) patients with serous cystadenocarcinomas had detectable serum PLAP (Table I). In the malignant disease group, patients with epithelial tumours had a greater incidence of circulating PLAP (22/62, 36%) than those with non-epithelial tumours (1/5, 20%) (Table I). There was no significant association between the levels of circulating PLAP and clinical staging (Fisher's exact test, $P > 0.05$), although stage I disease had a lower incidence of serum PLAP than more advanced carcinoma (Table II). For 12 patients with no known ovarian disease, and whose ovaries had been checked by laparoscopy, there was no detectable circulating PLAP (i.e. $< 0.1\, U\, l^{-1}$).

PLAP could be detected in trace amounts in soluble extracts of normal and benign ovarian

**Table I** PLAP in sera of patients with ovarian tumours

| | *Incidence (i.e. $\geq 0.1\, U\, l^{-1}$ PLAP)* | *Range of ($U\, l^{-1}$ PLAP)* |
|---|---|---|
| *Malignant epithelial tumours:* | | |
| Serous cystadenocarcinoma | 12/37 | 0.1–4.0 |
| Mucinous cystadenocarcinoma | 2/6 | 0.8–0.9 |
| Clear cell carcinoma | 0/1 | — |
| Endometrioid carcinoma | 4/8 | 0.1–1.2 |
| Mesonephroid carcinoma | 0/2 | — |
| Undifferentiated carcinoma | 4/8 | 0.1–4.7 |
| *Malignant non-epithelial tumours:* | | |
| Malignant teratoma | 0/1 | — |
| Granulosa cell tumour | 1/3 | 0.6 |
| Mixed germ cell tumour | 0/1 | — |
| *Non-malignant epithelial tumours:* | | |
| Serous cystadenoma | 1/9 | 0.3 |
| Mucinous cystadenoma | 3/7 | 0.1–0.5 |
| Endometrioma | 0/2 | — |
| *Non-malignant non-epithelial tumours:* | | |
| Cystic teratoma | 0/1 | — |
| Corpus luteum | 0/3 | — |

**Table II** PLAP in ovarian carcinoma of epithelial origin

| *Serum PLAP* | *Stage I* (%) | *Stage II* (%) | *Stage III* (%) | *Stage IV* (%) | *Recurrence* (%) |
|---|---|---|---|---|---|
| Positive ($\geq$0.1 U $l^{-1}$) | 1/6 (17) | 2/5 (40) | 11/31 (35) | 6/14 (43) | 1/6 (17) |
| Negative (<0.1 U $l^{-1}$) | 5/6 (83) | 3/5 (60) | 20/31 (65) | 8/14 (57) | 5/6 (83) |

tissues by EIA using H317, whereas PLAP was detected at increased levels in soluble extracts from malignant ovarian tissues (Table III). However, within the numbers of available tissues, there was no statistically significant difference ($P > 0.05$, Wilcoxon rank test) between the malignant and either the benign or normal group. Furthermore, no quantitative correlation could be found between tissue extract and circulating PLAP levels.

**Table III** PLAP in soluble ovarian tissue extracts

| *Tissue source* | *No. tested* | *PLAP level (U kg⁻¹ wet weight tissue)* mean | *(range)* |
|---|---|---|---|
| Normal ovaries | 6 | 6.6 | (<0.3–32.4) |
| Benign ovarian disease | 8 | 3.4 | (<0.3–10.0) |
| Malignant ovarian disease | 6 | 12.8 | (1.2–53.6) |

**Figure 1** Mucinous ovarian cystadenocarcinoma tissue stained with H317 mAb for PLAP in a PAP-immunoperoxidase technique and showing staining of perinuclear and basal areas of the tumour cells (arrows). Section counterstained with haematoxylin. ×600.

*Immunohistology*

A proportion of epithelial mucinous tumours, both benign and malignant, clearly stained for PLAP with H317. Thus, 3/7 (43%) of the benign cystadenomas and 1/3 (33%) of the cystadenocarcinomas showed definite reactivity with H317. The staining was mainly perinuclear and in the basal part of the tumour cells, especially close to the basement membrane, for PLAP-positive mucinous tumours (Figure 1). Foci of unstained tumour cells could be seen even in tissues where most tumour cells were strongly stained by H317. No distinctive morphological features could be determined in PLAP-negative tumour areas to differentiate them from the clearly PLAP-positive tumour areas.

A proportion of epithelial serous tumours also showed strong staining of both histologically benign (1/5; 20%) and malignant (3/11; 27%) tumour tissues. In the PLAP-positive benign tumours, staining occurred predominantly close to the apical membrane of these cells while, in the malignant tumours, cytoplasmic staining was more diffusely prominent in addition to apical staining (Figure 2). Clear cell carcinomas showed a variable staining pattern with 2/5 tumour tissues giving a strong positive cell membrane reaction with H317 (Figure 3), whereas 3 were negative.

The intensity of immunohistological staining for PLAP in ovarian tumours could not be correlated with clinical staging. Analysis of the available data on individual patients demonstrated that clearly positive immunohistological staining for PLAP showed some association with soluble tissue extract PLAP positivity, although immunohistology was less sensitive (Table IV).

**Figure 2** Papillary ovarian carcinoma tissue stained with H317 mAb for PLAP in a PAP-immunoperoxidase technique and showing supranuclear cytoplasmic staining (arrow). Section counterstained with haematoxylin. ×600.

**Figure 3** Clear cell ovarian carcinoma tissue stained with H317 mAb for PLAP in a PAP-immunoperoxidase technique and showing cell membrane staining (arrow). Section counterstained with haematoxylin. ×600.

**Table IV** Comparison of serum PLAP, tissue extract PLAP and immunohistology for PLAP in ovarian tumours

| *Serum* ($\geq 0.1\ U l^{-1}$ *PLAP*) | *Tissue extract* ($\geq 0.3\ U kg^{-1}$ *PLAP*) | *Immunohistology* | *No. of cases* ($n=14$) |
|---|---|---|---|
| + | + | + | 5/14 |
| − | + | + | 3/14 |
| − | + | − | 3/14 |
| − | − | − | 3/14 |
| − | − | + | 0/14 |
| + | − | − | 0/14 |
| + | − | + | 0/14 |
| + | + | − | 0/14 |

### Discussion

The H317 mAb has been used to investigate the production and release of PLAP by ovarian tumours using EIA and immunohistological techniques. This mAb, which reacts with most of the genetic phenotypes of PLAP, has been shown to recognise tumour-derived PLAP (McLaughlin *et al.*, 1982, 1983; McDicken *et al.*, 1983). In the present study, circulating PLAP was detected in over 30% of all patients with ovarian tumours. Circulating PLAP has previously been reported in ovarian cancer patients at an incidence ranging from 15% to 65% (Fishman *et al.*, 1975; Kellen *et al.*, 1976; Benham *et al.*, 1978; Haije *et al.*, 1979). This may reflect differences in patient selection as well as sensitivity and specificity of assay for PLAP. We have detected circulating PLAP in 34% of patients with malignant disease, while the incidence of circulating PLAP was 23% in those with benign tumours. Circulating PLAP levels did not appear to correlate with stage of disease or tumour burden, agreeing with a recent study by Doellgast & Homesley (1984). However, in the absence of disease, incidence of circulating PLAP in control sera was 0% in EIA using H317 contrasting with 31% in the study of Doellgast & Homesley (1984). This is probably a consequence of the H317-based assay not detecting the smoking-associated form of PLAP found in some normal sera (McLaughlin *et al.*, 1984*b*).

Such use of mAb specific for PLAP in EIA offers an improvement on assays based on heat stability of PLAP or polyclonal antisera which may have cross-reactivities for other AP isoenzymes. However, there is some limitation associated with the use of mAbs in that their fine specificity may sometimes result in a lack of reactivity with occasional forms of PLAP. H317, for example, is known (McLaughlin *et al.*, 1984*a*) not to react with the Nagao tumour-derived form which may occur in some ovarian carcinomas (Benham *et al.*, 1978). Nevertheless, single tissues can express both Regan-like and Nagao-like PLAP forms concomitantly (McLaughlin *et al.*, 1984*a*) and most PLAP-positive ovarian tumours co-express both Regan and Nagao tumour-derived PLAP types (McLaughlin *et al.*, unpublished). Hence, it is likely that there is no marked underestimation of PLAP positivity in ovarian carcinoma using H317.

PLAP could be clearly localized in ovarian tumour tissue using the H317 mAb in an immunoperoxidase staining technique on routinely fixed tissues, as also recently reported using a separate mAb (NDOG2) on frozen ovarian cancer tissue (Sunderland *et al.*, 1984). The distribution of PLAP differed between serous and mucinous tumours, with the isoenzyme being mainly detectable towards the luminal surface of serous epithelial cells whereas it was mainly detectable in the perinuclear cytoplasm and towards the base of mucinous cells. Diffuse cytoplasmic staining was the predominant finding in the more malignant tumours. Unstained tumour cells occurred more frequently in the less differentiated tissues, and in anaplastic tumours large areas were negative for PLAP using H317. A similar juxtaposition of staining and non-staining malignant cells within the same tumour tissue has previously been noted (McDicken *et al.*, 1983). It is possible that tumour cells express PLAP only at certain stages in the cell cycle (Fishman & Singer, 1975).

Levels of PLAP detected in ovarian tumour tissue extracts by H317 in EIA were variable, ranging up to $53\,U\,kg^{-1}$ wet weight tissue, and did not correlate with circulating PLAP levels. This discrepancy between tumour tissue and circulating PLAP levels has been reported previously in ovarian carcinoma (Benham *et al.*, 1978). PLAP has also been detected in various normal tissue extracts (Goldstein *et al.*, 1982; Millan *et al.*, 1982), including non-malignant ovarian tissues (McLaughlin *et al.*, 1984*a*). Since PLAP is not detected in the sera of healthy individuals using the EIA based on H317 (McLaughlin *et al.*, 1983, 1984*b*), this suggests that the cellular release of PLAP may determine its subsequent detection in circulation more than the tumour load or rate of PLAP production; cell membrane damage or cell death may be required before significant PLAP is released to the circulation. This is relevant to the finding of circulating PLAP in benign ovarian disease and also in cigarette smokers (Maslow *et al.*, 1983; Tonik *et al.*, 1983; McLaughlin *et al.*, 1984*b*). In these cases, serum PLAP may result from damage to organs and subsequent release of endogenous PLAP.

In conclusion, PLAP has been detected in the serum and tissue of a significant proportion of patients with ovarian tumours using sensitive and specific assays based on a mAb, H317. Detection of circulating PLAP in this serological assay is a marker of the presence of an ovarian tumour in over 30% of women presenting with an abdominal mass. Furthermore, this H317-based EIA does not detect the non-tumour, smoking-associated form(s) of PLAP found in some healthy individuals. In addition, our results have indicated the usefulness of H317 for the tissue localization of PLAP-producing tumour cells.

This work was supported by the North West Cancer Research Fund and the Imperial Cancer Research Fund.

### References

BENHAM, F.J., POVEY, M.S. & HARRIS, H. (1978). Placental-like alkaline phosphatase in malignant and benign ovarian tumours. *Clin. Chim. Acta*, **86**, 201.

BHATTACHARYA, M. & BARLOW, H. (1979). Tumour markers for ovarian cancer. *Int. Adv. Surg. Oncol.*, **2**, 155.

DOELLGAST, G.J. & HOMESLEY, D. (1984). Placental-type alkaline phosphatase in ovarian cancer fluids and tissues. *Obstet. Gynecol.*, **63**, 324.

FISHMAN, W.H. & SINGER, R.M. (1975). Placental alkaline phosphatase: Regulation of expression in cancer cells. *Ann. N.Y. Acad. Sci.*, **259**, 261.

FISHMAN, W.H., INGLIS, N.R., VAITUKAITIS, J. & STOLBACH, L.L. (1975). Regan isoenzyme and human chorionic gonadotrophin in ovarian cancer. *Natl Cancer Inst. Monogr.*, **42**, 63.

GOLDSTEIN, D.J., ROGERS, C. & HARRIS, H. (1982). A search for trace expression of placental-like alkaline phosphatase in non-malignant human tissues: demonstration of its occurrence in lung, cervix, testis and thymus. *Clin. Chim. Acta*, **125**, 63.

HAIJE, W.G. (1982). Biochemical markers in ovarian cancer: possibilities and limitations. *Ann. Clin. Biochem.*, **19**, 258.

HAIJE, W.G., MEERWALDT, J.H., TALERMAN, A & 5 others (1979). The value of a sensitive assay of carcinoplacental alkaline phosphatase (CPAP) in the follow-up of gynaecological cancers. *Int. J. Cancer*, **24**, 288.

JOHNSON, P.M., CHENG, H.M., MOLLOY, C.M. STERN, C.M.M. & SLADE, M.B. (1981). Human trophoblast-specific surface antigens identified using monoclonal antibodies. *Am. J. Reprod. Immunol.*, **1**, 246.

JOHNSON, P.M. & MOLLOY, C.M. (1983). Localization in human term placental bed and amniochorion of cells bearing trophoblast antigens identified by monoclonal antibodies. *Am. J. Reprod. Immunol.*, **4**, 33.

KELLEN, J.A., BUSH, R.S. & MALKIN, A. (1976). Placental-like alkaline phosphatase in gynecological cancers. *Cancer Res.*, **36**, 269.

McDICKEN, K.W., STAMP, G.H., McLAUGHLIN, P.J. & JOHNSON, P.M. (1983). Expression of human placental-type alkaline phosphatase in primary breast cancer. *Int. J. Cancer*, **32**, 205.

McLAUGHLIN, P.J., CHENG, H.M., SLADE, M.B. & JOHNSON, P.M. (1982). Expression on cultured human tumour cell lines of placental trophoblast membrane antigens and placental alkaline phosphatase defined by monoclonal antibodies. *Int. J. Cancer*, **30**, 21.

McLAUGHLIN, P.J., GEE, H. & JOHNSON, P.M. (1983). Placental-type alkaline phosphatase in pregnancy and malignancy plasma: specific estimation using a monoclonal antibody in a solid phase enzyme immunoassay. *Clin. Chim. Acta*, **130**, 199.

McLAUGHLIN, P.J., TRAVERS, P.J., McDICKEN, I.W. & JOHNSON, P.M. (1984*a*). Demonstration of placental and placental-like alkaline phosphatases in non-malignant human tissue extracts using monoclonal antibodies in an enzyme immunoassay. *Clin. Chim. Acta*, **137**, 341.

McLAUGHLIN, P.J., TWIST, A.M., EVANS, C.C. & JOHNSON, P.M. (1984*b*). Serum placental-type alkaline phosphatase in cigarette smokers. *J. Clin. Pathol.*, **37**, 826.

MASLOW, W.C., MUENZCH, H.A., AZAMA, F. & SCHNEIDER, A.S. (1983). Sensitive fluorimetry of heat-stable alkaline phosphatase (Regan enzyme) activity in serum from smokers and non-smokers. *Clin. Chem.*, **29**, 260.

MILLAN, J.L., ERIKSSON, A. & STIGBRAND, T. (1982). A possible new locus of alkaline phosphatase expressed in human testis. *Hum. Genet.*, **62**, 293.

STIGBRAND, T., MILLAN, J.L. & FISHMAN, W.H. (1983). The genetic basis of alkaline phosphatase isozyme expression. *Curr. Top. Biol. Med. Res.*, **6**, 93.

SUNDERLAND, C.A., DAVIES, J.O. & STIRRAT, G.M. (1984). Immunohistology of normal and ovarian cancer tissue with a monoclonal antibody to placental alkaline phosphatase. *Cancer Res.*, **44**, 4496.

TONIK, S.E., ORTMEYER, A.E., SHINDELMAN, J.E. & SUSSMAN, H.H. (1983). Elevation of serum placental alkaline phosphatase levels in cigarette smokers. *Int. J. Cancer*, **31**, 51.

WEI, S.C. & DOELLGAST, G.J. (1981). Immunochemical studies of human placental-type variants of alkaline phosphatase. *Eur. J. Biochem.*, **118**, 39.